# One Day in Denmark: Comparison of Phenotypic and Genotypic Antimicrobial Susceptibility Testing in Bacterial Isolates From Clinical Settings

**DOI:** 10.3389/fmicb.2022.804627

**Published:** 2022-06-10

**Authors:** Ana Rita Rebelo, Valeria Bortolaia, Pimlapas Leekitcharoenphon, Dennis Schrøder Hansen, Hans Linde Nielsen, Svend Ellermann-Eriksen, Michael Kemp, Bent Løwe Røder, Niels Frimodt-Møller, Turid Snekloth Søndergaard, John Eugenio Coia, Claus Østergaard, Henrik Westh, Frank M. Aarestrup

**Affiliations:** ^1^National Food Institute, Technical University of Denmark, Kongens Lyngby, Denmark; ^2^Department of Bacteria, Parasites and Fungi, Statens Serum Institut, Copenhagen, Denmark; ^3^Department of Clinical Microbiology, Herlev and Gentofte Hospital, Herlev, Denmark; ^4^Department of Clinical Microbiology, Aalborg University Hospital, Aalborg, Denmark; ^5^Department of Clinical Medicine, Aalborg University, Aalborg, Denmark; ^6^Department of Clinical Microbiology, Aarhus University Hospital, Aarhus, Denmark; ^7^Department of Clinical Microbiology, Odense University Hospital, Odense, Denmark; ^8^Department of Clinical Microbiology, Slagelse Hospital, Slagelse, Denmark; ^9^Department of Clinical Microbiology, Rigshospitalet, Copenhagen, Denmark; ^10^Department of Clinical Microbiology, Hospital of Southern Jutland, Sønderborg, Denmark; ^11^Department of Clinical Microbiology, Hospital of South West Jutland, Esbjerg, Denmark; ^12^Department of Clinical Microbiology, Vejle Hospital, Vejle, Denmark; ^13^Department of Clinical Microbiology, Hvidovre Hospital, Copenhagen University Hospital – Amager and Hvidovre, Hvidovre, Denmark; ^14^Department of Clinical Medicine, University of Copenhagen, Copenhagen, Denmark

**Keywords:** whole-genome sequencing (WGS), antimicrobial resistance (AMR), antimicrobial resistance genes (ARGs), genotype, phenotype, concordance, *in silico* antibiogram

## Abstract

Antimicrobial susceptibility testing (AST) should be fast and accurate, leading to proper interventions and therapeutic success. Clinical microbiology laboratories rely on phenotypic methods, but the continuous improvement and decrease in the cost of whole-genome sequencing (WGS) technologies make them an attractive alternative. Studies evaluating the performance of WGS-based prediction of antimicrobial resistance (AMR) for selected bacterial species have shown promising results. There are, however, significant gaps in the literature evaluating the applicability of WGS as a diagnostics method in real-life clinical settings against the range of bacterial pathogens experienced there. Thus, we compared standard phenotypic AST results with WGS-based predictions of AMR profiles in bacterial isolates without preselection of defined species, to evaluate the applicability of WGS as a diagnostics method in clinical settings. We collected all bacterial isolates processed by all Danish Clinical Microbiology Laboratories in 1 day. We randomly selected 500 isolates without any preselection of species. We performed AST through standard broth microdilution (BMD) for 488 isolates (*n* = 6,487 phenotypic AST results) and compared results with *in silico* antibiograms obtained through WGS (Illumina NextSeq) followed by bioinformatics analyses using ResFinder 4.0 (*n* = 5,229 comparisons). A higher proportion of AMR was observed for Gram-negative bacteria (10.9%) than for Gram-positive bacteria (6.1%). Comparison of BMD with WGS data yielded a concordance of 91.7%, with discordant results mainly due to phenotypically susceptible isolates harboring genetic AMR determinants. These cases correspond to 6.2% of all isolate-antimicrobial combinations analyzed and to 6.8% of all phenotypically susceptible combinations. We detected fewer cases of phenotypically resistant isolates without any known genetic resistance mechanism, particularly 2.1% of all combinations analyzed, which corresponded to 26.4% of all detected phenotypic resistances. Most discordances were observed for specific combinations of species-antimicrobial: macrolides and tetracycline in streptococci, ciprofloxacin and β-lactams in combination with β-lactamase inhibitors in *Enterobacterales*, and most antimicrobials in *Pseudomonas aeruginosa*. WGS has the potential to be used for surveillance and routine clinical microbiology. However, in clinical microbiology settings and especially for certain species and antimicrobial agent combinations, further developments in AMR gene databases are needed to ensure higher concordance between *in silico* predictions and expected phenotypic AMR profiles.

## Introduction

Antimicrobial susceptibility testing (AST) is one of the main components of clinical microbiology diagnostics. With the emergence of new antimicrobial resistance (AMR) mechanisms and multi- or pan-drug resistant organisms, it becomes increasingly important to ensure that adequate antimicrobial susceptibility profiles are available in a fast and accurate manner, leading to proper interventions, drug prescriptions, and therapeutic success (Cassini et al., 2019).

Broth microdilution (BMD) is the AST method endorsed by the European Committee on Antimicrobial Susceptibility Testing (EUCAST), performed according to the recommendations from the International Organization for Standardization [ISO 20776-1:2006 (International Organization for Standardization, 2006) and ISO 20776-1:2019 (International Organization for Standardization, 2019)]. BMD requires relatively simple techniques and limited costs for well-developed laboratories (Jorgensen and Ferraro, 2009; Benkova et al., 2020). However, the reproducibility of BMD and other phenotypic AST protocols, such as disk diffusion, remains a concern. Small variations in, for example, operators’ methodology, laboratory materials and reagents, machinery, and culture conditions may translate into different results and respective interpretations (Wiegand et al., 2008). Furthermore, the agreement between results derived from different phenotypic methods and cross-interpretation of the same is not perfect (Jean et al., 2017; Matuschek et al., 2018; Mojica et al., 2020).

Studies have been performed to compare classical phenotypic AST results with *in silico* antibiograms obtained through whole-genome sequencing (WGS) technologies. Notably, EUCAST performed an in-depth review of phenotype–genotype AMR concordance in important human pathogens, concluding that for certain bacterial groups (such as *Enterobacteriaceae* and staphylococci), results have been promising with high levels of concordance, while for other species (e.g., *Pseudomonas aeruginosa*) prove much more difficult to interpret (Ellington et al., 2017). These findings are corroborated by other projects that have shown good concordance between WGS-based antimicrobial susceptibility predictions and minimum inhibitory concentration (MIC) determinations for *Enterobacterales* (Zankari et al., 2012; Stoesser et al., 2013; Pecora et al., 2015; Clausen et al., 2016; Do Nascimento et al., 2017; Shelburne et al., 2017; Ruppé et al., 2020), enterococci, staphylococci, and streptococci (Aanensen et al., 2016; Metcalf et al., 2016, 2017; Hammerum et al., 2017; Mason et al., 2018; Stewart et al., 2020), while remaining more variables for *P. aeruginosa* (Kos et al., 2015; Subedi et al., 2018; Cortes-Lara et al., 2021). The main caveat of these and other similar projects is their focus on selected bacterial species, thus not reflecting the diversity observed during routine testing (Tagini and Greub, 2017; Su et al., 2018). Studies evaluating WGS data of non-preselected bacterial isolates collected in a clinical setting exist but are comprised of small collections or mainly focus on species identification and epidemiological analyses, without exploring the use of genomic data for AMR prediction (Long et al., 2013; Hasman et al., 2014; Roach et al., 2015; Galata et al., 2019).

This study was conducted to compare AST results obtained with reference laboratory MIC determinations and WGS predictions, based on a random selection of clinical isolates obtained during a single day from all clinical microbiological laboratories in Denmark.

## Materials and Methods

### Bacterial Isolates

We collected all clinically relevant isolates (*n* = 2,073) processed by the 11 Danish Clinical Microbiology Laboratories (DCM) (Herlev and Gentofte Hospital, Herlev; Hvidovre Hospital, Hvidovre; Nykøbing F. Sygehus, Nykøbing F; Odense Universitetshospital, Odense; Rigshospitalet, København; Slagelse Sygehus, Slagelse; Sydvestjysk Sygehus, Esbjerg; Sygehus Lillebælt, Vejle; Sygehus Sønderjylland, Sønderborg; Aalborg Universitetshospital, Aalborg; and Aarhus Universitetshospital, Skejby) on January 10, 2018, as previously described (Rebelo et al., 2022). According to the accompanying metadata, 2,024 of these corresponded to bacterial isolates. Before any analyses or laboratory proceedings, we performed a random selection of 500 isolates using the “=RAND” function on Microsoft Excel to attribute a random number to each of the 2,024 presumptive bacterial isolates, locking the obtained values, sorting them by value from lowest to highest, and selecting the first 500.

### Phenotypic Antimicrobial Susceptibility Testing

Antimicrobial susceptibility testing was performed at European Union Reference Laboratory for Antimicrobial Resistance (EURL-AR), at the Technical University of Denmark (DTU) by BMD using Thermo Scientific™ Sensititre™ panels, in particular GN3F (*n* = 266), GPALL1F (*n* = 35), EUSTAPF (*n* = 103), STP6F (*n* = 70), HPB1 (*n* = 14), and FRCOL (*n* = 265, of which 251 were used in combination with GN3F and 14 in combination with HPB1), and performed through agar dilution for anaerobic bacteria (*n* = 7) according to international standards [ISO 20776-1:2006 (International Organization for Standardization, 2006), EUCAST guidelines (European Committee on Antimicrobial Susceptibility Testing, 2017), and CLSI M11-A8 (Clinical and Laboratory Standards Institute, 2012)] and according to the manufacturer’s specifications.

Interpretation of MIC results was performed using EUCAST clinical breakpoints version 12.0. When different breakpoints existed for “meningitis” and “indications other than meningitis,” the latter was applied due to the absence of isolates from cerebrospinal fluid in this collection. When breakpoints only referred to urinary tract infections, they were still applied to allow for phenotype–genotype comparison. For easier description and discussion of results, we grouped the “susceptible, standard dosing regimen” (S) and “susceptible, increased exposure” (I) categories under the term “susceptible,” as currently recommended by EUCAST (European Committee on Antimicrobial Susceptibility Testing, 2021). All cut-off values are described in Supplementary Table 3.

### Whole-Genome Sequencing-Based Antimicrobial Susceptibility Testing

Whole-genome sequencing and bioinformatics analyses were performed at DTU. Genomic DNA was extracted from all bacterial isolates using the Easy-DNA™ Kit (Invitrogen, Carlsbad, CA, United States), and DNA concentrations were determined using the Qubit™ dsDNA high-sensitivity (HS) and/or broad-range (BR) assay kits (Invitrogen, Carlsbad, CA, United States). Genomic DNA was prepared for Illumina paired-end sequencing using the Illumina (Illumina, Inc., San Diego, CA, United States) NexteraXT^®^ DNA Library Prep Reference Guide (Document #15031942, v03, February 2018) and NextSeq System Denature and Dilute Libraries Guide (Document #15048776, v03, April 2018). The libraries were sequenced using the Illumina NextSeq 500 platform. The raw reads were *de novo* assembled using the Centre for Genomic Epidemiology FoodQCPipeline^1^ for assembly and quality control. Quality thresholds were set at maximum 500 contigs per genome and maximum 0.5 million base pairs of deviation from the expected genome size. Species identification was performed as previously described (Rebelo et al., 2022) through matrix-assisted laser desorption/ionization – time-of-flight mass spectrometry (MALDI-TOF MS), KmerFinder^2^ (Larsen et al., 2014; Clausen et al., 2018), and rMLST^3^ (Jolley et al., 2012).

*In silico* AST was performed using ResFinder 4.0^4^ (Bortolaia et al., 2020). The program was run in batch by grouping the isolates per species, using the Danish National Supercomputer for Life Sciences^5^ and using the default threshold values found in the corresponding online tool (minimum accepted alignment of 60% and minimum accepted identity of 90%). Results were manually curated for trimethoprim/sulfamethoxazole in all relevant species (because resistance genes for each antimicrobial are provided separately using the ResFinder 4.0 tool).

We classified WGS results as “resistant” when one or several antimicrobial resistance genes (ARGs) or chromosomal point mutations (PMs) were identified by ResFinder and allocated as the mechanism of AMR to that antimicrobial, and as “susceptible” when no ARG or PM was found.

Raw sequence data have been submitted to the European Nucleotide Archive^6^ under study accession no.: PRJEB37711. A complete list of genomic sequence data is available in the Supplementary Material.

### Comparison of Phenotype and Genotype

We compared MIC results and WGS results for all bacterial species-antimicrobial combinations where EUCAST clinical breakpoints were available and included in the Sensititre™ panel range (excluding intrinsic resistances), while simultaneously being present in the ResFinder 4.0 database (*n* = 5,229).

The antimicrobials present in the BMD panels but missing from the ResFinder 4.0 database, and thus automatically excluded from the comparison, were as follows: ampicillin/sulbactam, cefaclor, cefazolin, cefpodoxime, ceftaroline, cefuroxime, clarithromycin, daptomycin, levofloxacin, moxifloxacin, nitrofurantoin, norfloxacin, oxacillin, sparfloxacin, and telavancin. The antimicrobials present in the panels and in the ResFinder database but not analyzed due to lack of breakpoints or due to these values being outside of the panel range are available on a species-antimicrobial basis in the Supplementary Material.

## Results

### Bacterial Isolates

Of the 500 randomly selected bacterial isolates, two were non-viable from the beginning of laboratory work (presumptive *Neisseria gonorrhoeae* isolates) and one died during laboratory work (presumptive *Aerococcus* sp. isolate). For one isolate, no MIC values were obtained (*Anaerococcus hydrogenalis*), and for another, they were not interpretable (undetermined species). Seven anaerobic isolates were excluded due to failure to obtain MIC values in the admitted range for the ATCC control strains.

**FIGURE 1:**
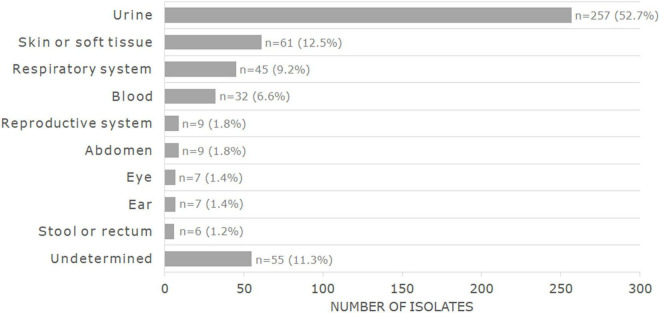
Distribution by sample source of the 488 bacterial isolates analyzed in this study.

The MIC and WGS results were obtained and interpreted for a total of 488 bacterial isolates, of which the main species were *Escherichia coli* (*n* = 171, 35%) and *Staphylococcus aureus* (*n* = 89, 18.2%). The complete distribution of isolates by genera and species can be found in Table 1 and in Supplementary Table 1. Most isolates had been recovered from urine samples (*n* = 257, 52.7%) or skin/soft tissue samples (*n* = 61, 12.5%). The complete distribution of the isolated source is found in Figure 1.

**TABLE 1:** Distribution by genera of the 488 bacterial isolates analyzed in this study.

Genera	Number of isolates	Percentage
*Escherichia*	171	35.0
*Staphylococcus*	103	21.1
*Streptococcus*	55	11.3
*Klebsiella*	34	7.0
*Enterococcus*	32	6.6
*Pseudomonas*	21	4.3
*Haemophilus*	14	2.9
*Moraxella*	10	2.0
*Proteus*	10	2.0
*Enterobacter*	10	2.0
*Citrobacter*	10	2.0
*Aerococcus*	5	1.0
*Corynebacterium*	3	0.6
*Stenotrophomonas*	2	0.4
*Serratia*	2	0.4
*Erwinia*	1	0.2
*Morganella*	1	0.2
*Providencia*	1	0.2
*Raoultella*	1	0.2
*Salmonella*	1	0.2
*Yersinia*	1	0.2
**Grand total**	**488**	**100**

### Classical Methods for Antimicrobial Susceptibility Testing

For the 488 bacterial isolates, the total number of obtained MIC results was 10,874, including cefoxitin, gentamicin, and streptomycin screens and *D*-tests. The total number of MIC values interpreted was 6,487 (Tables 2, 3). The remaining MIC values were not interpreted due to lack of breakpoints or due to intrinsic resistance of the respective species to the respective antimicrobial but are provided in the Supplementary Material. The screens and *D*-tests were interpreted for relevant species. In summary, from the 6,487 isolate-antimicrobial combinations interpreted, 91% (*n* = 5,901) corresponded to susceptible phenotypes (S or I) and 9% (*n* = 586) to resistance phenotypes (R).

**TABLE 2:** Phenotypic antimicrobial susceptibility testing (AST) results obtained through broth microdilution (BMD) for the main Gram-positive bacterial taxa in this study.

Bacteria	Antimicrobials	Nr. of tests	Nr. (%) of susceptible^1^ isolates	Nr. (%) of resistant isolates
Staphylococci (*n* = 103)	Erythromycin	103	97 (94.2%)	6 (5.8%)
	Clindamycin	103	100 (97.1%)	3 (2.9%)
	Tetracycline	103	96 (93.2%)	7 (6.8%)
	Trimethoprim/sulfamethoxazole	103	100 (97.1%)	3 (2.9%)
	Gentamicin	103	99 (96.1%)	4 (3.9%)
	Tobramycin	103	96 (93.2%)	7 (6.8%)
	Fusidic acid	102	80 (78.4%)	22 (21.6%)
	Linezolid	103	102 (99%)	1 (1%)
	Teicoplanin	103	102 (99%)	1 (1%)
	Vancomycin	103	103 (100%)	0 (0%)
	Rifampicin	103	101 (98.1%)	2 (1.9%)
	Cefoxitin	94	92 (97.9%)	2 (2.1%)
	Ceftaroline	90	90 (100%)	0 (0%)
	Daptomycin	103	102 (99%)	1 (1%)
	Levofloxacin	103	95 (92.2%)	8 (7.8%)
	Norfloxacin	90	83 (92.2%)	7 (7.8%)
	Moxifloxacin	103	94 (91.3%)	9 (8.7%)
	**Total**	**1,625**	**1,549 (95.3%)**	**76 (4.7%)**
Streptococci (*n* = 55)	Erythromycin	51	39 (76.5%)	12 (23.5%)
	Azithromycin	51	40 (78.4%)	11 (21.6%)
	Clindamycin	55	50 (90.9%)	5 (9.1%)
	Tetracycline	51	32 (62.7%)	19 (37.3%)
	Tigecycline	44	44 (100%)	0 (0%)
	Trimethoprim/sulfamethoxazole	51	51 (100%)	0 (0%)
	Chloramphenicol	51	50 (98%)	1 (2%)
	Penicillin	55	55 (100%)	0 (0%)
	Ceftriaxone, cefotaxime, cefepime, ertapenem, and meropenem	55	55 (100%)	0 (0%)
	Linezolid	51	51 (100%)	0 (0%)
	Vancomycin	55	55 (100%)	0 (0%)
	Daptomycin	44	44 (100%)	0 (0%)
	Levofloxacin	51	50 (98%)	1 (2%)
	**Total**	**665**	**616 (92.6%)**	**49 (7.4%)**
Enterococci (*n* = 32)	Quinupristin/dalfopristin	9	8 (88.9%)	1 (11.1%)
	Tigecycline	32	32 (100%)	0 (0%)
	Ampicillin	32	23 (71.9%)	9 (28.1%)
	Linezolid	32	32 (100%)	0 (0%)
	Vancomycin	32	32 (100%)	0 (0%)
	Levofloxacin	32	21 (65.6%)	11 (34.4%)
	Nitrofurantoin	23	23 (100%)	0 (0%)
	**Total**	**192**	**171 (89.1%)**	**21 (10.9%)**
*Corynebacterium* spp. (*n* = 3)	Penicillin and clindamycin	6	0 (0%)	6 (100%)
	Linezolid, vancomycin, and rifampicin	9	9 (100%)	0 (0%)
	Ciprofloxacin and moxifloxacin	6	4 (66.7%)	2 (33.3%)
	Tetracycline	3	2 (66.7%)	1 (33.3%)
	**Total**	**24**	**15 (62.5%)**	**9 (37.5%)**
*Aerococcus* spp. (*n* = 5)	Penicillin, meropenem, vancomycin, and levofloxacin	20	20 (100%)	0 (0%)
	**Grand total**	**2,526**	**2,371 (93.9%)**	**155 (6.1%)**

*^1^Susceptible isolates include those classified as susceptible, standard dosing regimen (S) and susceptible, increased exposure (I) according to the European Committee on Antimicrobial Susceptibility Testing (EUCAST) clinical breakpoints version 12.0.*

**TABLE 3:** Phenotypic AST results obtained through BMD for the main Gram-negative bacterial taxa in this study.

Bacteria	Antimicrobials	Nr. of tests	Nr. (%) of susceptible^1^ isolates	Nr. (%) of resistant isolates
*Enterobacterales* (*n* = 243)	Ampicillin	183	113 (61.7%)	70 (38.3%)
	Ticarcillin/clavulanic acid, ampicillin/sulbactam	471	358 (76%)	113 (24%)
	Cefazolin	205	168 (82%)	37 (18%)
	Cefuroxime	216	181 (83.8%)	35 (16.2%)
	Ceftazidime and ceftriaxone	486	458 (94.2%)	28 (5.8%)
	Cefepime	243	238 (97.9%)	5 (2.1%)
	Meropenem	243	243 (100%)	0 (0%)
	Aztreonam	243	227 (93.4%)	16 (6.6%)
	Ciprofloxacin	242	214 (88.4%)	28 (11.6%)
	Trimethoprim/sulfamethoxazole	243	198 (81.5%)	45 (18.5%)
	Gentamicin	242	231 (95.5%)	11 (4.5%)
	Amikacin	242	238 (98.3%)	4 (1.7%)
	Colistin	229	226 (98.7%)	3 (1.3%)
	**Total**	**3,488**	**3,093 (88.7%)**	**395 (11.3%)**
*Pseudomonas aeruginosa* (*n* = 21)	Piperacillin/tazobactam	21	20 (95.2%)	1 (4.8%)
	Ticarcillin/clavulanic acid	21	4 (19%)	17 (81%)
	Ceftazidime	21	21 (100%)	0 (0%)
	Cefepime	21	20 (95.2%)	1 (4.8%)
	Meropenem	21	21 (100%)	0 (0%)
	Aztreonam	21	20 (95.2%)	1 (4.8%)
	Ciprofloxacin	21	17 (81%)	4 (19%)
	Amikacin	21	21 (100%)	0 (0%)
	Colistin	21	21 (100%)	0 (0%)
	**Total**	**189**	**165 (87.3%)**	**24 (12.7%)**
*Haemophilus influenzae* (*n* = 14)	Ampicillin	14	9 (64.3%)	5 (35.7%)
	Amoxicillin/clavulanic acid, ampicillin/sulbactam	28	25 (89.3%)	3 (10.7%)
	Cefuroxime, cefixime, ceftriaxone, and cefepime	56	54 (96.4%)	2 (3.6%)
	Imipenem and meropenem	28	28 (100%)	0 (0%)
	Chloramphenicol, tetracycline, and levofloxacin	42	42 (100%)	0 (0%)
	Trimethoprim/sulfamethoxazole	14	12 (85.7%)	2 (14.3%)
	**Total**	**182**	**170 (93.4%)**	**12 (6.6%)**
*Moraxella catarrhalis* (*n* = 10)	Cefuroxime, ceftriaxone, cefotaxime, cefepime, ertapenem, meropenem, erythromycin, azithromycin, tetracycline, trimethoprim/sulfamethoxazole	100	100 (100%)	0 (0%)
*Stenotrophomonas maltophilia* (*n* = 2)	Trimethoprim/sulfamethoxazole	2	2 (100%)	0 (0%)
	**Grand total**	**3,961**	**3,530 (89.1%)**	**431 (10.9%)**

*^1^Susceptible isolates include those classified as susceptible, standard dosing regimen (S) and susceptible, increased exposure (I) according to the EUCAST clinical breakpoints version 12.0.*

Gram-positive bacteria presented lower proportions of phenotypical resistance (6.1%) than Gram-negative bacteria (10.9%).

### Comparison of Phenotype and Genotype

**FIGURE 2:**
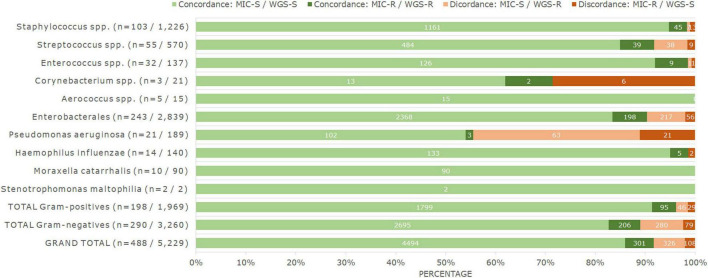
Percentage and number of genotype–phenotype concordances and discordances observed for all bacteria analyzed in this study, when comparing MIC and WGS results. Numbers in brackets for each taxon correspond to the number of isolates, and to the number of isolate-antimicrobial combinations tested. Numbers on top of the bars correspond to the respective number of results. MIC-S cases correspond to S and I phenotypes according to EUCAST clinical breakpoints v12.0, and MIC-R cases correspond to R phenotypes according to the same guideline. WGS-S correspond to cases where no acquired ARGs nor chromosomal PMs have been detected, and WGS-R correspond to cases where they have.

Out of the 5,229 isolate-antimicrobial combinations for which MIC and WGS data were compared, 434 (8.3%) had discordances in phenotypic and genotypic antimicrobial susceptibility profiles. The remaining 4,795 combinations (91.7%) showed a concordance between phenotype and genotype (Figure 2). Of the observed discordances, 75 were observed in Gram-positive bacteria, which corresponded to 3.8% of all 1,969 combinations analyzed in those isolates. Among Gram-negative bacteria, there were 359 discordances, or 11% of the 3,260 combinations analyzed in those taxa (Figure 2).

The discordant cases were not equally distributed. For 326 isolate-antimicrobial combinations, ARGs or PMs were detected but the isolate was phenotypically susceptible (major errors). This number corresponded to 6.2% of all combinations analyzed and to 6.8% of all phenotypically susceptible combinations (*n* = 4,820). The remaining 108 discordances were phenotypically resistant isolates in which no genetic determinants of AMR were detected (very major errors), corresponding to 2.1% of all combinations analyzed and 26.4% of all the detected phenotypic resistances (*n* = 409) (Figure 2).

In staphylococci, we analyzed 103 isolates with 12 antimicrobials, yielding a total of 1,226 isolate-antimicrobial combinations. In total, 1.6% (*n* = 20) of results were discordant. Notably, 25% of these discordances occurred for fusidic acid (*n* = 5 or 4.9% of 102 combinations analyzed for this antimicrobial). Furthermore, we observed one, two, or three discordances for all remaining antimicrobials tested except vancomycin and erythromycin, which presented none.

For streptococci, we analyzed 55 isolates with 15 antimicrobials (a total of 570 combinations). The worst phenotype–genotype concordance was observed for macrolide antimicrobials. Of all isolate-macrolide combinations (*n* = 102), 34.3% (*n* = 35) corresponded to discordances, mainly due to phenotypic susceptibility with the presence of ARGs (*n* = 30). Furthermore, we detected eight discordances for tetracycline (15.7% of tetracycline results). These two classes were responsible for 91.5% of all discordances observed in streptococci. The remaining discordances were found in clindamycin (*n* = 3 or 5.5% of results for clindamycin) and chloramphenicol (*n* = 1 or 2% of chloramphenicol results). In total, 8.2% (*n* = 47) of results were discordant.

In enterococci, we analyzed the results of 32 isolates and four antimicrobials, as well as quinupristin/dalfopristin results in nine *Enterococcus faecium* isolates, with a total of 137 isolate-antimicrobial combinations. Discordances were only observed for quinupristin/dalfopristin (*n* = 2 or 22.2% of quinupristin/dalfopristin results), with one resistant isolate missing dalfopristin resistance genes and one susceptible (I) isolate harboring quinupristin and dalfopristin resistance genes. Although this percentage is high, in total, only those two results were discordant (1.5% of all results).

Of the remaining Gram-positive bacteria, no discordances were found in the 15 isolate-antimicrobial combinations analyzed for the five isolates of *Aerococcus* spp. The three isolates of *Corynebacterium* spp. were analyzed with seven antimicrobials (*n* = 21 combinations), and we observed six discordances (28.6% of all results). All these discordances corresponded to phenotypically resistant isolates with no known resistance genes, for penicillin (*n* = 3, 100%), clindamycin (*n* = 2, 66.6%), and ciprofloxacin (*n* = 1, 33.3%). Tetracycline, vancomycin, rifampicin, and linezolid results were concordant in this genus.

In *Enterobacterales*, 243 isolates were analyzed with 12 antimicrobials (except when certain species were intrinsically resistant or lacked breakpoints), yielding a total of 2,839 combinations. In total, 9.6% (*n* = 273) of results were discordant. Ciprofloxacin concordance was poor, with discordances observed in 28.5% of results for this antimicrobial, which corresponded to 25.3% of all detected discordances. The remaining discordances were mainly observed for the β-lactam antibiotics (*n* = 187) and together were responsible for 68.5% of all discordances observed in *Enterobacterales*. In particular, combinations of penicillins with β-lactamase inhibitor results were discordant in 28.8% of cases (*n* = 70 of 243 results), followed by cephalosporins (12.3% or *n* = 90 of 729 results) and aztreonam (12.1% or *n* = 26 of 243 results). These issues were mainly due to phenotypic susceptibility profiles with the presence of ARGs (139 out of 186 discordances in these antimicrobials). However, no discordances were observed for meropenem and only one (0.5%) for ampicillin. Only 17 other cases of discordances were observed for this bacterial group, with no other antimicrobial exceeding 2.5% of discordant results.

*Pseudomonas aeruginosa* had the highest percentage of discordant results. Concordance was analyzed for 21 isolates with nine antimicrobials (*n* = 189 combinations), and 44.4% of results showed discordance between phenotype and genotype (*n* = 84). Several antimicrobials showed over 50% of phenotype–genotype discordance, in particular ceftazidime (100%), cefepime (95.2%), meropenem (61.9%), and ciprofloxacin (52.4%), with genetic determinants present in phenotypically susceptible isolates, and ticarcillin/clavulanic acid (81%) with resistant isolates harboring no known genetic AMR determinants. The remaining β-lactam antibiotics, namely, piperacillin/tazobactam and aztreonam also had poor results, with 4.8% of discordances each. Only amikacin and colistin showed full genotype–phenotype concordance.

Of the 140 combinations analyzed for *Haemophilus influenzae* (14 isolates with 10 antimicrobials), two (1.4%) were discordant, observed for trimethoprim/sulfamethoxazole. Due to the small number of isolates, these corresponded to 14.3% of the results for this antimicrobial. All other combinations (*n* = 138) were concordant.

No discordances were found for *Moraxella catarrhalis* (10 isolates with nine antimicrobials, yielding 90 combinations) nor *Stenotrophomonas maltophilia* (two isolates with one antimicrobial).

All individual and grouped genotype–phenotype comparison results are available as Supplementary Material.

## Discussion

In this study, we analyzed both phenotypic and genotypic AMR profiles of a random subset of clinically relevant bacterial isolates obtained from an original collection containing all isolates processed during one day in all Danish Clinical Microbiology Laboratories, without any *a priori* selection of bacterial species nor biological sample type.

Only 9% (*n* = 586) of all phenotypic AST results (*n* = 6,487) determined by the standard BMD corresponded to phenotypical resistance, which can be considered as a low occurrence of AMR when compared with results observed in similar settings, such as other countries in the European Union (European Centre for Disease Prevention and Control [ECDC], 2020). The AMR profiles observed in this study are mostly in agreement with what has been observed in surveillance results for urine and/or invasive isolates analyzed in the last decade, country-wide (Statens Serum Institut and National Food Institute, 2018, 2019, 2020). Noteworthy observations are that *Staphylococcus epidermidis* and *Streptococcus agalactiae* were the species with the highest prevalence of AMR in the *Staphylococcus* and *Streptococcus* genera, respectively (Supplementary Tables 2,3). Furthermore, the occurrence of ampicillin resistance in *Enterococcus* spp. corresponded to 100% of resistance in *E. faecium* isolates and full susceptibility in *Enterococcus faecalis* (Supplementary Tables 2,3). In both *Enterobacterales* and *P. aeruginosa*, resistance to combinations of β-lactams with β-lactamase inhibitors and to ciprofloxacin was high, and other prevalent resistance phenotypes were detected (Table 3 and Supplementary Tables 2,3).

The main focus of this study was the comparison of BMD AST results with *in silico* antibiograms obtained through WGS followed by bioinformatics analysis. WGS-based AMR predictions were discordant from BMD results in 8.3% of all species-antimicrobial combinations analyzed, from which 6.2% corresponded to major errors and 2.1% to very major errors. Most of these discordances were restricted to particular species-antimicrobial class combinations, in particular, macrolides and tetracycline in streptococci, penicillins in association with β-lactamase inhibitors, ciprofloxacin in *Enterobacterales*, and most antimicrobials in *P. aeruginosa*.

Most of the streptococci-macrolide discordances (*n* = 30) were due to the presence of the ARG *mre(A)* in phenotypically susceptible isolates. The gene was also observed in phenotypically resistant isolates but in combination with *erm*, *mef*, or *msr* genes, which are associated with macrolide resistance (Garland et al., 2011; Metcalf et al., 2017). As such, it appears that *mre(A)* by itself is unable to increase the macrolides’ MIC above resistance thresholds in streptococci, in opposition to what had been previously suggested (Clarebout et al., 2001). Tetracycline resistance genotypic determinants *tet(M)* and *tet(O)* were more heterogeneously distributed: of the 21 isolates harboring these ARGs 16 were phenotypically resistant, but five were susceptible. Furthermore, three isolates with tetracycline MIC values in the resistant range presented no known genes associated with resistance, indicating that further studies are necessary to elucidate the correlation between genetic determinants and phenotypes, as well as to discover other genetic mechanisms potentially responsible for tetracycline resistance in streptococci. The poor correlation between the presence or absence of *tet* genes and phenotypic tetracycline-resistance has been observed in other instances (Stewart et al., 2020).

In *Enterobacterales*, the discordances observed in piperacillin/tazobactam were equally distributed between the presence of ARGs in phenotypically susceptible isolates (*n* = 35) and the opposite situation of phenotypic resistance without any known AMR determinants (*n* = 35). Predicting the activity of these combinations through genomic analyses has proven difficult in the past, as alterations in gene expression, mutations in promoter regions, and number of gene copies, among other factors, can contribute to altered phenotypes while being difficult to detect through these WGS methods (Stoesser et al., 2013; Schechter et al., 2018; Ruppé et al., 2020). Ciprofloxacin discordances were mainly due to the presence, in phenotypically susceptible isolates, of single PMs in gyrase and topoisomerase genes and to the presence of *oqx* genes. The *oqxA* and *oqxB* ARGs increase MIC when compared with values observed in wild-type isolates but are not always responsible for an increase that would lead to a classification of clinical resistance. However, they can lead to such an increase and thus cannot be discarded from *in silico* antibiograms (Hansen et al., 2007; Wong et al., 2015). Furthermore, it has been previously shown that the number of gyrase and topoisomerase point mutations is correlated with the observed increase in quinolone-MIC values: accumulation of more mutations leads to higher MICs (Vila et al., 1994; Everett et al., 1996; Sáenz et al., 2003; Hopkins et al., 2005). As such, we would suggest that it is necessary to use a different approach for predicting ciprofloxacin resistance than the usual detection of the presence/absence of genotypic determinants. Although the absence of PMs or ARGs is a good predictor of susceptibility, algorithms should consider both the type and number of mutations detected in each isolate, as well as the association with acquired ARGs when attempting to predict phenotypic resistance.

In *P. aeruginosa*, discordances were abundant and distributed throughout several different antimicrobial classes. Against what should be expected according to previous studies, the presence of β-lactamases from the *bla*_OXA_ and *bla*_PAO_ families and of the *crpP* gene did not correlate to phenotypic resistance to β-lactam drugs and ciprofloxacin, respectively (Rodríguez-Martínez et al., 2009;Chávez-Jacobo et al., 2018; Subedi et al., 2018; Madaha et al., 2020). AMR is often exacerbated or mediated by complex genetic mechanisms leading to overexpression of *ampC*-encoded β-lactamases and efflux pumps in this species, which confounds genotypic detection of resistance without resorting to, for example, transcriptomics or proteomics analyses (Tam et al., 2007; Lister et al., 2009; Cabot et al., 2011).

We have shown that WGS can potentially be applied as a diagnostic method for AST in clinical microbiology settings. There are clear limitations when considering certain species-antimicrobial combinations where mechanisms related to increased gene expression are involved, but the phenotype–genotype concordance for most isolates processed in the clinical microbiology laboratories was very high. However, as previously pointed out, this study was conducted in a collection presenting relatively low percentages of phenotypic AMR. We observed very major errors in 26.4% of all phenotypically resistant isolate-antimicrobial combinations, but these cases only represented 2.1% of all our results. Thus, phenotype–genotype concordance might be lower in settings with a higher prevalence of AMR; therefore, a future perspective is to employ this methodology of data comparison in bacterial collections with more challenging phenotypes. This approach will elucidate which species-antimicrobial combinations should be further investigated regarding currently unknown genetic determinants of AMR. For these combinations, analyzing large datasets of MIC distributions and comparing those with the genome sequences can reveal new ARGs or PMs of interest, and their contribution to phenotypic resistance profiles can be then confirmed *in vitro* through, for example, transformation experiments (Hadjirin et al., 2021). This procedure can also be used to confirm if specific genetic determinants that apparently do not confer phenotypic resistance can be removed from bioinformatics databases, despite previous literature suggesting that association. Machine learning approaches can also be employed to identify further genetic candidates associated with AMR (Peiffer-Smadja et al., 2020; Anahtar et al., 2021).

Besides the need to improve our understanding of phenotypic–genotypic correlations for the discordant cases and curate bioinformatics databases according to the state of the art, it is necessary to create an adequate framework that allows for the use of these results in a clinical setting. In this study, the choice of clinical breakpoints referring to “indications other than meningitis” and the inclusion of breakpoints exclusive for uncomplicated urinary tract infection were made to allow the inclusion of larger amounts of data for phenotype and genotype comparison, due to the most common sample sources observed in this collection of bacterial isolates. However, in a clinical setting, the difference in breakpoints cannot be ignored, and data analysis systems must take into account sample sources and other important clinical manifestations of the disease. One potential solution is to associate warning messages to antimicrobial-species combinations for which there is a meningitis breakpoint, when the breakpoint only applies to urinary tract infection or other specific situations. Furthermore, current data are insufficient to effectively predict differences between S and I categories through genotypic characterization, thus systems must also warn users when the susceptibility categories include both S and I ranges (or only I ranges). These details must be confirmed and updated any time the breakpoints are revised, similarly to the current practice of updating the interpretation of phenotypic results. In this study, we have provided the MIC distributions in addition to their interpretation, so it is possible to review phenotype–genotype concordance when breakpoints are revised, or new breakpoints are included.

Implementation of *in silico* prediction of AMR profiles in clinical settings must be gradual. Laboratories should start with benchmarking their WGS and bioinformatics approaches, by employing the technology in parallel with their currently used diagnostics methods for well-described bacterial species, from uncomplicated infections, and with simple expected phenotypes. Only after optimizing all processes related to WGS, bioinformatics analysis, and data management, should laboratories consider an exclusive WGS-based characterization of isolates (Florian Fricke and Rasko, 2014; Gargis et al., 2016). Depending on the specific local bacterial epidemiology patterns, laboratories can choose to proceed with the inclusion of all isolates processed in their settings, or apply WGS technologies only in selected cases (such as for species included in national surveillance systems, species associated with frequent nosocomial or community outbreaks, or species with emerging epidemiological relevance in neighboring settings).

It is furthermore important to note that a major caveat of performing genotypic predictions of antimicrobial susceptibility profiles is the lack of detection of new resistance determinants. Bioinformatics tools screen for ARGs or chromosomal PMs included in their databases and are thus unable to detect any unknown AMR determinants. Therefore, it is likely that some national laboratories must continue to perform sentinel or routine phenotypic AST so that variations in incidence or prevalence of phenotypic resistance can serve as a warning that new mechanisms of resistance might have been acquired or might be emerging. Together, these limitations make it unlikely that phenotypic methods can be completely replaced by *in silico* antibiograms. However, the possibility of applying WGS technologies to other steps of the clinical diagnostics pipeline (such as species identification, serotyping, and sequence typing, among others) and for infection control purposes (through phylogenetic or cluster analyses), the constantly decreasing cost of sequencing machines and reagents and the ease of storing, processing, and transferring data make it an attractive option in clinical settings that should be further explored and optimized (Didelot et al., 2012; Deurenberg et al., 2017; Parcell et al., 2021).

## Data Availability Statement

The datasets presented in this study can be found in online repositories. The names of the repository/repositories and accession number(s) can be found in the article/Supplementary Material.

## Author Contributions

All authors contributed to the conception and design of the study. DH, HN, SE-E, MK, BR, NF-M, TS, JC, CØ, and HW provided the bacterial isolates and the respective metadata, as well as routine clinical diagnostics results. AR performed antimicrobial susceptibility testing through broth microdilution, DNA extraction, whole-genome sequencing, bioinformatics analysis, data analysis, and wrote the first draft of the manuscript. All authors contributed to manuscript revision, read, and approved the submitted version.

## Conflict of Interest

The authors declare that the research was conducted in the absence of any commercial or financial relationships that could be construed as a potential conflict of interest. The reviewer PD declared a shared parent affiliation with the HW to the handling editor at the time of review.

## Publisher’s Note

All claims expressed in this article are solely those of the authors and do not necessarily represent those of their affiliated organizations, or those of the publisher, the editors and the reviewers. Any product that may be evaluated in this article, or claim that may be made by its manufacturer, is not guaranteed or endorsed by the publisher.

## Footnotes

1
https://bitbucket.org/genomicepidemiology/foodqcpipeline/src/master/

2
https://cge.food.dtu.dk/services/KmerFinder/

3
https://pubmlst.org/rmlst/

4
https://cge.food.dtu.dk/services/ResFinder/

5
https://www.computerome.dk

6
http://www.ebi.ac.uk/ena
